# The sequence of events in six years of a myopic traction maculopathy

**DOI:** 10.3205/oc000256

**Published:** 2025-08-26

**Authors:** Prabu Baskaran, Bharg Kariya, Anand Rajendran

**Affiliations:** 1 Aravind Eye Hospital, Chennai, India

**Keywords:** myopia, maculopathy, traction maculopathy, myopic detachment

## Abstract

**Purpose:**

To describe the sequence of events in a case of high myope with myopic traction maculopathy.

**Methods:**

Our female patient who is a high myope developed myopic choroidal neovascular membrane (CNVM), for which she received three anti-vascular endothelial growth factor injections (VEGF). It was scarred for a while. Later she developed myopic foveoschisis with macular detachment which progressed over a period with deterioration of vision.

**Results:**

She underwent pars plana vitrectomy with silicone oil tamponade. Oil removal was done eight months later. At the last follow up visit, her macula was flat with stable vision.

**Conclusion:**

Myopic traction maculopathy (MTM) is a challenging case to manage with its myriad of presentations and complex pathology.

## Introduction

Myopic macular foveoschisis was first reported on optical coherence tomography (OCT) by Takano et al. in 1999 [1]. However, the term did not gain much approval as it was not a true schisis and also has varied presentations. Pannozzo et al. proposed to unify all these pathologies under one entity and coined the term myopic traction maculopathy (MTM) [2]. The spectrum of presentation includes vitreo-macular traction, foveal retinal detachment and macular hole. Our case shows the evolution of multiple MTM pathologies over a span of 6 years along with its medical and surgical management. 

## Case description

**Figure 1:**
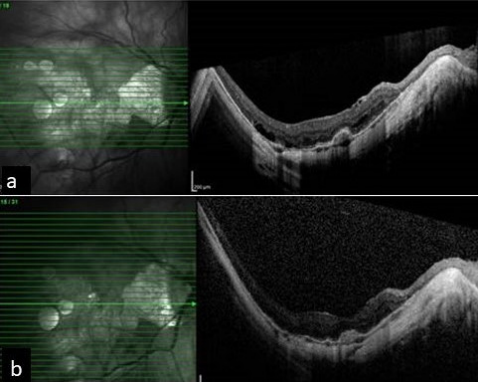
a) OCT image showing sub-foveal myopic CNVM at presentation. b) OCT image showing regressed CNVM after 3 anti VEGF injections

**Figure 2:**
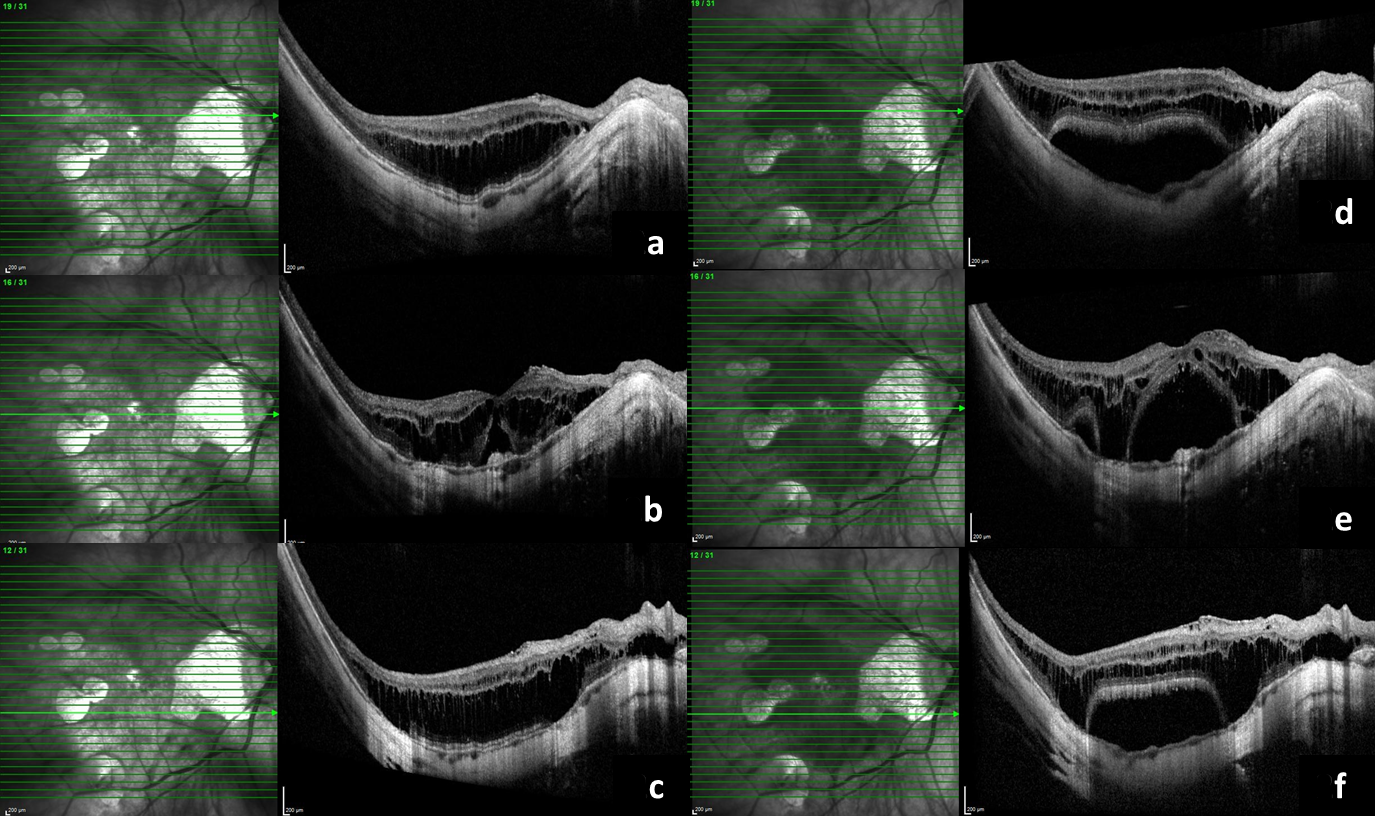
a, b, c) OCT scans taken above, at, and below the level of fovea, respectively, showing early sub-foveal detachment; d, e, f) OCT scans taken above, at, and below the level of fovea, respectively, showing the progressive foveal detachment involving entire macula

**Figure 3:**
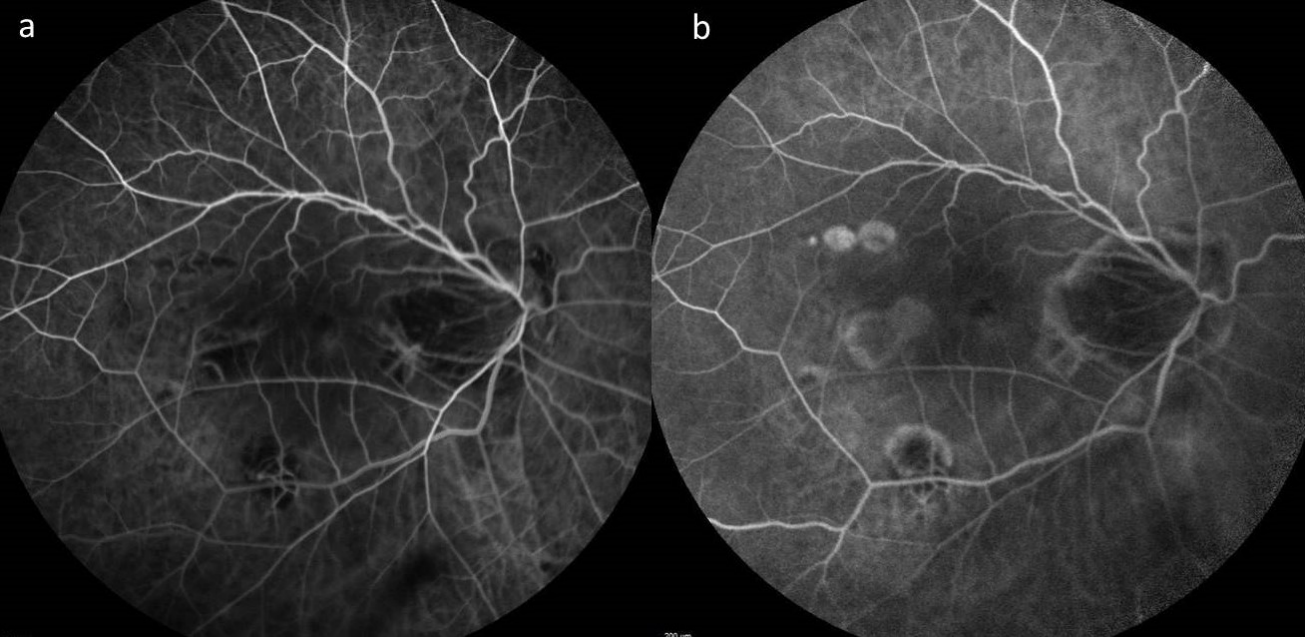
Fundus fluorescein angiography of the patient performed when there was progression of sub-retinal fluid, did not show any reactivation, a) early phase, b) late phase

**Figure 4:**
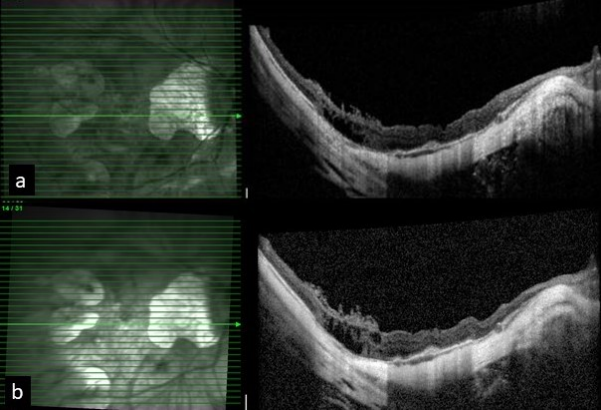
a) Settled foveal detachment after vitrectomy at one month post-operative visit. b) Stable macula at five month follow up visit post SOR

A 55-year-old female presented to us for the first time in 2013 for a routine check-up. The best corrected visual acuity (BCVA) in both eyes was 6/9 (Snellen visual acuity). Axial length in the right eye (RE) was 31.00 mm and in the left eye (LE) was 28.73 mm. The patient was on routine follow-up since then. In July 2016, she presented with complaints of RE blurred vision since the last two months. On examination, RE BCVA had deteriorated from 6/9 to 6/60.The fundus examination showed altered foveal reflex, which on OCT (768x496 , 30 degrees, 8.8 mm, Spectralis, Heidelberg Engineering, Germany) revealed a myopic sub-foveal choroidal neovascular membrane (CNVM) (Figure 1a (Fig. 1)). Left eye findings were status quo. She received three monthly anti-vascular endothelial growth factor (VEGF) intravitreal injections for RE. Her RE BCVA improved to 6/9 along with regression of the myopic CNVM (Figure 1b (Fig. 1)). In May 2018, the patient again presented with diminished vision in the right eye, with BCVA deteriorated from 6/9 to 6/18. OCT revealed the presence of a shallow sub-foveal neurosensory detachment in addition to macular schisis. The patient was kept under close observation. The sub foveal neurosensory detachment kept increasing steadily over a period of 3 months (Figure 2a, b, c, d, e, f (Fig. 2)) and the BCVA deteriorated to 6/36. Fundus fluorescein angiography did not reveal any reactivation of CNVM (Figure 3 (Fig. 3)). A 25 gauge pars plana vitrectomy, internal limiting membrane (ILM) peeling, sub-retinal fluid drainage (SRFD) and silicon oil injection (5,000 centistokes) was performed to arrest the progression of macular detachment. The SRFD was achieved by creating a small drainage retinotomy at the margin of the detachment, near the supero-temporal arcade and barraged. The rationale of SRFD was to achieve rapid resolution of such a voluminous SRF. Macula was settled on first post-operative day. One month later, the patient continued to have a stable retina with attached macula (Figure 4a (Fig. 4)). The BCVA improved to 6/24 at one month follow up visit. Silicone oil removal (SOR) was done 8 months later. At the final visit (5 months after SOR) the patient had a stable retina with flat macula with 6/24 BCVA (Figure 4b (Fig. 4)).

## Discussion

Pannozzo and Mercanti proposed that the earlier terminology macular foveoschisis is a misnomer and coined the term MTM in view of the absence of an absolute scotoma [2]. The authors suggested three possible primary traction mechanisms involved in MTM. They are namely: 1) anterior traction by the vitreous, 2) tangential traction by the internal limiting membrane (ILM), and 3) posterior traction by staphyloma. Shimada et al. classified MTM based on the area of schisis (S) (S0 – absent, S1 – extrafoveal, S2 – foveal, S3 – foveal but not entire macula, S4 – entire macula) [3]. They observed that S4 lesions tend to progress over time unlike other types. Our case involved the entire macula, i.e. stage S4. Shimada et al. reported an increased preponderance of advanced MTM stages in patients with myopic CNVM [3].

Wong et al. reported myopic CNVM incidence of 5–11% in patients with high myopia [4]. In patients with high myopia, continued axial elongation leads to breaks in Bruch’s membrane in the retroequatorial region [5] and reduced subfoveal choroidal thickness leading to progression of myopic maculopathy and CNVM [6]. 

The management of MTM is mostly conservative due to its complex nature and unpredictable surgical outcome. The debatable point in such cases is when to operate. However, presence of foveal retinal detachment warrants an early vitrectomy [7]. The choice of the procedure depends on the primary traction mechanism. The anterior traction is primarily relieved by pars plana vitrectomy (PPV) and the tangential traction is relieved by ILM peeling. In most cases, the posterior traction mechanism is less significant and hence less commonly addressed. However, it has been suggested that significant posterior traction due to a large staphyloma may need a macular buckle with or without PPV [8]. Macular buckle was not needed in our case as the posterior staphyloma was not significant. Also macular buckle is not available in our country. Shimada et al. suggested the role of fovea-sparing ILM peeling in MTM [9]. The rationale for sparing the fovea while peeling ILM is to reduce the risk of occurrence of post-operative macular hole. Conventional ILM peeling may de-roof the bridging tissue over the fovea and can result in a macular hole. In a cohort of 41 eyes, Chang et al. observed that patients with foveal detachment along with MTM tend to have better final visual outcome than MTM alone or MTM with macular hole [10]. It is unclear whether the sub retinal fluid (SRF) needs to be drained or whether an endo-tamponade is necessary or not. However, we opted to drain the SRF and inject high density silicone oil (5,000 centistokes) to ensure early and continued stability. MTM can have a variety of presentations as seen in our case. The progression of MTM is slow and might take many years. Hence long-term periodic monitoring is necessary to understand the evolution of the disease. We report this case to document how MTM morphed from one form to another over a span of six years’ time. Our case is unique as it is, to the best of our knowledge, the first reported instance of successful surgical management of a case of MTM developing after the resolution of myopic CNVM with anti-VEGF therapy. At last visit, the patient remained stable with neither recurrence of CNVM nor SRF.

## Notes

### Competing interests

The authors declare that they have no competing interests.
